# Octopamine and tyramine dynamics predict learning rate phenotypes during associative conditioning in honey bees

**DOI:** 10.1126/sciadv.aea8433

**Published:** 2026-02-11

**Authors:** Lester P. Sands, Hong Lei, Seth R. Batten, Alec Hartle, Terry Lohrenz, Leonardo Barbosa, Dan Bang, Peter Dayan, William M. Howe, Brian H. Smith, Pendleton R. Montague

**Affiliations:** ^1^ Fralin Biomedical Research Institute at VTC, Virginia Tech, Roanoke, Virginia 24016, USA.; ^2^ School of Life Sciences, Arizona State University, Tempe, Arizona 85281, USA.; ^3^ School of Neuroscience, Virginia Tech, Blacksburg, Virginia 24061, USA.; ^4^ Center of Functionally Integrative Neuroscience, Aarhus University, Aarhus, Denmark.; ^5^ Max Planck Institute for Biological Cybernetics, Tubingen, Germany.; ^6^ University of Tubingen, Tubingen, Germany.; ^7^ Department of Physics, Virginia Tech, Blacksburg, Virginia 24061, USA.

## Abstract

Biogenic amines are fundamental for physiological homeostasis and behavioral control in both vertebrates and invertebrates. Monoamine neurotransmitters released in target brain regions conjointly regulate adaptive learning and plasticity. However, our understanding of these multianalyte mechanisms remains nascent, in part due to limitations in measurement technology. Here, during associative conditioning in honey bees, we concurrently tracked subsecond fluctuations in octopamine, tyramine, dopamine, and serotonin in the antennal lobe, where plasticity influences odorant representations. By repeatedly pairing an odorant with subsequent sucrose delivery, we observed individual differences in the conditioned response to odor, which occurred after a variable number of pairings (learners) or not at all (non-learners). The distinction between learners and non-learners was reflected in neurotransmitter responses across experimental conditions. The speed of learning, the number of pairings prior to a proboscis extension reflex, could be predicted from monoamine opponent signaling (octopamine-tyramine), from both the first presentation of the odorant alone, prior to any pairing with sucrose, and the first conditioned response to the odorant, coming after a number of sucrose pairings. These results suggest that monoamine signaling phenotypes may relate directly to the now widely reported socially relevant genetic differences in honey bee learning.

## Teaser

Subsecond octopamine and tyramine opponent signaling in antennal lobe predicts the rate of odorant conditioning in honey bees.

## INTRODUCTION

Honey bee foragers are remarkably good at learning the associations between floral cues and the nectar or pollen rewards that their colonies need to survive in rapidly changing landscapes (*1*). As a result, honey bees have been developed as a model for understanding cognition in natural and laboratory settings (*2*). Nonassociative and associative conditioning in honey bees are typically studied in a controlled setting using the proboscis extension response (PER) conditioning procedure, which involves pairing an odor with sucrose reinforcement such that the bees develop associatively conditioned responses to the odor (*3*). Classical conditioning of the PER can occur quickly, with some bees only taking a few pairings of an odor conditioned stimulus (CS) with food reinforcement, and the memory is retained for several days thereafter. However, individual bees differ in whether or how fast they learn (*4*), and individual differences revealed in PER are correlated with worker foraging decisions in a colony-level foraging strategy (*5*).

PER conditioning has been used successfully to uncover neural correlates of the cellular and molecular substrates of memory (*2*, *6*). Using this behavioral protocol, different biogenic amines have been implicated in driving excitatory or inhibitory neural plasticity underlying the behavior. For instance, pioneering work in the 1990’s demonstrated a role for the monoamine octopamine (OA) in mediating honey bee PER conditioning, showing that electrophysiological excitation of the OA-releasing interneuron VUMmx1 of the subesophageal ganglion could substitute as a reinforcement signal during associative learning (*7*, *8*). Still, several different biogenic amines typically affect processing within subnetworks of the brain; for example, the VUMmx1 neuron must make tyramine (TYM) to make OA, and it probably releases both neurotransmitters in the antennal lobes and mushroom bodies (*9*, *10*). However, to date, it has remained difficult to further resolve the mechanistic roles of multiple biogenic amines during PER conditioning, due in part to a lack of direct, moment-to-moment estimates of neurotransmitter dynamics in the honey bee brain during associative learning.

Here, we used a machine learning–augmented electrochemistry method (voltammetry), which has been implemented in humans (*11*–*15*) and validated in vivo in rodents (*16*), to concurrently extract subsecond estimates of four neurotransmitters important for honey bee sensory processing and learning: dopamine (DA), serotonin (5HT), TYM, and OA. These monoamines play integral roles in physiological homeostasis, movement, and adaptive learning processes in invertebrates, with OA and TYM commonly understood as the invertebrate analogs to epinephrine and norepinephrine in the mammalian adrenergic system (*17*). We deployed this method in the antennal lobe of the brain during a standard PER odorant conditioning task, where the development of a PER is the behavioral readout of the conditioned response. The antennal lobes are the site of first-order synaptic interactions between olfactory sensory neurons from the antennae in the periphery and interneurons of the brain (*18*). Associative and nonassociative behavioral conditioning change the neural representations of odors in the antennal lobe to increase separation of odors that bees need to discriminate (*19*–*21*), at least in part through release of OA (*22*). Moreover, there is now evidence that OA and TYM may correlate, respectively, with excitatory (*8*, *22*) and inhibitory (*23*) behavioral conditioning and the foraging strategies they support (*24*). However, more generally, the nature of the phasic signaling activity and the potential computational roles of these various monoamines during honey bee associative learning remain unknown. Here, we show that the relative difference in OA and TYM levels may represent a neurochemical phenotype that determine the ability of individual honey bees to learn about the predictive nature of odorant cues.

## RESULTS

**Fig. 1.**
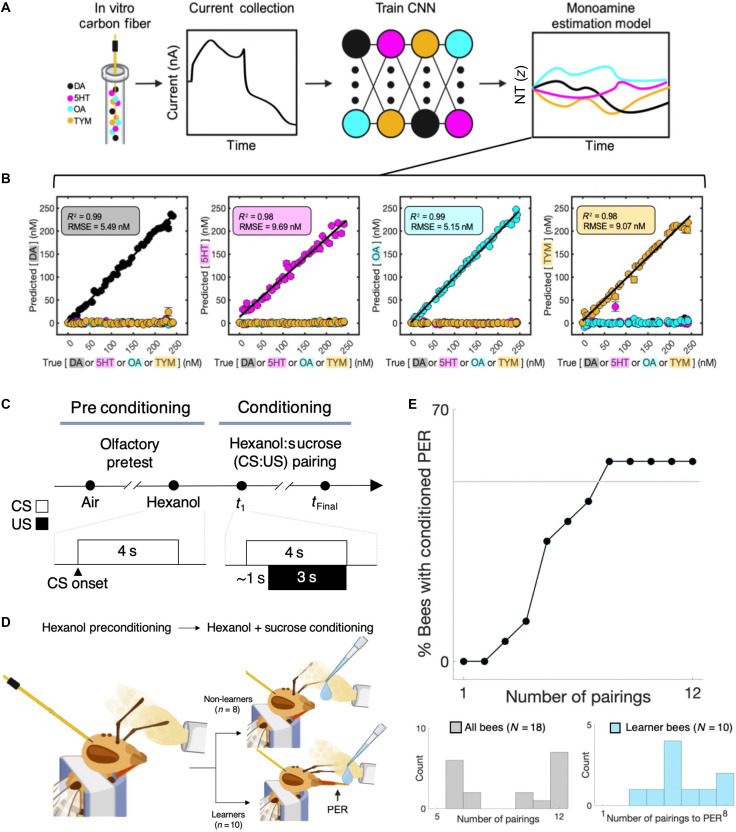
Monoamine vector estimation during honey bee odorant conditioning. (**A**) Diagram of machine learning–enhanced voltammetry procedure for monoamine concentration prediction. We constructed carbon fiber electrodes and exposed them to varying concentrations of DA, 5HT, OA, and TYM against a background of varying pH while using fast scan cyclic voltammetry (FSCV) to measure current responses to each combination. These responses were then used to train an ensemble of deep CNNs via cross-validation to generate a multianalyte concentration estimation model. (**B**) Performance of the trained ensemble. Results shown for out-of-sample predictions (nanomolar) for DA (black), 5HT (magenta), OA (cyan), and TYM (yellow), with each molecule shown with their mean ± one standard error of the mean (SEM) defined across the five different electrodes used in the experiments. RMSE, root mean square error. (**C**) Experimental timeline. Each experiment began with a preconditioning phase, consisting of olfactory stimulation trials [air (control) and hexanol], followed by an odorant conditioning phase, consisting of up to 12 hexanol:sucrose (CS:US) pairings. Insets depict the approximate timings of CS and US delivery across trial conditions; note that sucrose (US) delivery during conditioning trials occurred ~1 s after the hexanol puff. (**D**) After completing the conditioning phase, individual bees were classified as “learner” bees (*n* = 10) if they demonstrated a PER to the CS during conditioning, with the remainder classified as “non-learner” bees (*n* = 8). (**E**) Population curve of PER learning behaviors across conditioning trials for all bees (*N* = 18); bottom: distribution of the number of conditioning trials across all bees (left) and, for learner bees (right), the number of pairings before having PER to the CS (i.e., learning rate). Note that learner bees each had a variable number of trials from the PER trial to the final pairing.

We sought to record concurrent, subsecond changes in four monoamines during PER odorant conditioning using our validated, machine learning–enhanced approach to standard electrochemistry procedures. Figure 1 (A and B) shows the in vitro calibration protocol and performance of the method, which uses an ensemble of cross-validated deep convolutional neural network (CNN) models to generate monoamine concentration estimates from in vivo electrochemical recordings during honey bee behavioral conditioning Figure 1 (C and D) shows the elements of the conditioning paradigm and the behavioral measures that categorized bees as learners or non-learners based on development of a PER. A total of 18 bees were included in our study (see Materials and Methods for more details on bee selection and electrode placement; fig. S1), and of those 18 bees, 10 developed a PER (“learners”) after repeated pairings of an initially novel odorant (hexanol) with sucrose, whereas eight responded to the sucrose unconditioned stimulus (US) but never showed a PER to odor within the timeframe of the experiment (“non-learners”). The PER behavior itself can be treated as an all-or-none response in which the proboscis is extended in response to an odor or to antennal stimulation with a sucrose solution (see Materials and Methods). With repeated pairings of sucrose with an odor CS, the PER will occur in response to the odor alone (*3*). Within the learners, an individual bee’s learning rate, defined as the number of odor-sucrose pairings before the first extension of the proboscis to the conditioned odorant prior to delivery of sucrose reinforcement, varied between three and eight pairings (Fig. 1E).

Overall, across both preconditioning odorant exposure and odorant exposure during conditioning trials, we show that neurotransmitter fluctuations differed between learners and non-learners (tables S1 to S3), corroborating the observed behavioral differences between the groups. Therefore, we focused on individual differences in neurotransmitter response dynamics within learners for our primary analyses. For learners, the monoamine dynamics during the very first presentation of odor during the preconditioning phase (before any reinforcement), and during the conditioning trial where the bee first demonstrated a conditioned PER to the odor presentation, revealed a remarkable relationship to individual bee learning rates.

**Fig. 2.**
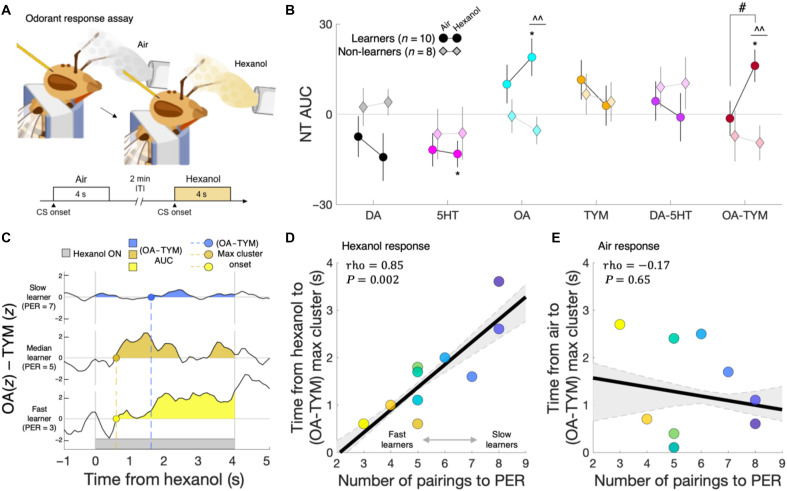
OA and TYM opponent response to hexanol preconditioning predicts PER learning rate. (**A**) Schematic of honey bee odorant response assay. Bees were exposed to 4 s of (control) air and hexanol across two trials, with each trial separated by 2 min. ITI, intertrial interval. (**B**) Response levels (AUCs) for neurotransmitters (NT) and opponent pairs across the odor stimulation conditions. Circles and diamonds represent mean group values; vertical lines depict SEM. *P < 0.05, one-sample *t* test; ^^*P* < 0.01, two-sample *t* test; #*P* < 0.05, paired *t* test. (**C**) Example of slow, median, and fast learner bees’ (*n* = 10) in vivo (OA-TYM) opponent response to hexanol, with positive AUC clusters highlighted and the onset of the maximum positive cluster indicated by the dashed line. (**D**) Correlation of individual learner bees’ (OA-TYM) maximum positive cluster onset time during hexanol exposure with their subsequent learning rate (trials to PER) during conditioning. (**E**) Correlation of learner bees’ (OA-TYM) maximum positive cluster onset time during control air puff with their learning rate. In both (D) and (E), the black line indicates the group-level correlation (rho) and the gray dashed lines and shaded region indicate the maximum and minimum of the (*k*-fold) bootstrapped group-level correlation.

During the preconditioning phase (Fig. 2), all bees were exposed on separate trials to a control air stream (fig. S2) and an air stream containing hexanol (fig. S3), which was used as the CS later in the experiment, as well as the other primary alcohol heptanol and 2-octanone. These three odorants are among several that have been widely used in honey bee PER conditioning (*25*), and the similarities in neural activity they elicit in the antennal lobe reflect similarities of the molecular structures. To investigate the computational roles of biogenic amines in guiding behavioral adaptations during associative learning (*26*), we analyzed the time series of OA, TYM, DA, and 5HT as well as two opponent pairs: DA-5HT (*27*) and OA-TYM. We evaluated the first pair, given the known roles for DA and 5HT opponency in mammalian behavioral control (*20*, *28*, *29*); we evaluated the latter pair because of their known involvement of OA and TYM in antagonistic regulation of insect behaviors (*17*). We computed the response level of each neurotransmitter and opponent pair by integrating the time series values within the 4-s olfactory stimulation period for each condition [i.e., area under the curve (AUC); Fig. 2B].

We first tested whether the response levels of biogenic amines or their opponent pairs were related to well-established differences in whether bees show a conditioned response to odor after its pairing with reinforcement. Linear modeling assessing the effects of group (learner or non-learner), odor (air or hexanol), and their interaction on neurotransmitter response levels revealed significant main group effects for DA, OA, and OA-TYM responses (table S1) [analysis of variance (ANOVA) main effect of group: DA, *F*(1,33) = 5.27, *P* = 0.028; OA, *F*(1,33) = 10.1, *P* = 0.003; OA-TYM, *F*(1,33) = 6.8, *P* = 0.013]. For non-learners, neurotransmitter and opponent pair response levels to air and hexanol were not significantly different from zero, and there were no differences between air and hexanol responses (Fig. 2B and table S2). In contrast, learners demonstrated significant OA, 5HT, and OA-TYM responses to hexanol, with a specific role for the OA-TYM opponency signal, which not only distinguished hexanol responses between learners and non-learners (table S1) but also distinguished air and hexanol responses within learners (Fig. 2B and table S2) (learner ANOVA main effect of odor: OA-TYM, *F*(1,19) = 4.57, *P* = 0.045; paired *t* test: *t*(18) = 2.2, *P* = 0.040). Given this finding, we sought to determine whether the hexanol neurotransmitter response levels were specific to this odor or generalized to other odors. To this end, we examined the representational similarity of air, hexanol, heptanol, and 2-octanone neurotransmitter response patterns (i.e., the six-vector of AUC values; fig. S4). Across all bees, we found significant similarity in monoamine profiles across all three odors (fig. S4), which indicates that changes in neurotransmitter responses during PER conditioning described below are state- or experience-dependent changes rather than being specific to one or another odorant.

We next evaluated whether neurotransmitter response levels to odor prior to conditioning might relate to the degree to which bees learned at different rates during conditioning. To this end, we regressed the learners’ neurotransmitter response levels to hexanol preconditioning against bees’ subsequent learning rates and found that the timing and magnitude of the OA-TYM opponent response to odor was predictive of the number of pairings eventually required for the bee to develop a PER (Fig. 2, C and D, and fig. S5). That is, learners that were fast to acquire a PER during conditioning demonstrated both a larger OA-TYM AUC response (fig. S5B) and an earlier onset of the maximum OA-TYM cluster (Fig. 2, C and D, and fig. S5A) in response to odor exposure preconditioning as compared to slower learners. These two measures, the OA-TYM response magnitude and timing, were significantly related to each other (fig. S5C), suggesting a step change–like functional response that was reflected in the group-averaged OA-TYM time series (fig. S3F). These measures were specific to odor sensation and not air stimulation (Fig. 2E and fig. S5D). Moreover, we found that this effect was absent for DA-5HT opponency (fig. S6), further emphasizing the specificity of the odor-evoked OA-TYM opponency as predictive of learning rate. Lastly, to investigate how learning rate might relate to latent patterns in the temporal dynamics of the neurotransmitter time series in response to hexanol, we performed a singular value decomposition (SVD) of bee monoamine dynamics in response to odor exposure preconditioning (fig. S7). This SVD revealed one singular vector with weights reflecting a similar step change or sign flip–like response pattern in the OA-TYM opponency signal (but not DA/5HT opponency), the loading of which was predictive of bee learning rate (fig. S7). The encoding of the learning rate carried by the moment-by-moment opponent OA-TYM signal occurred on the first presentation of hexanol and before any pairings with sucrose had occurred, which suggests that bees’ neurochemical dynamics before learning affect whether or how they attend to and learn about stimulus associations.

**Fig. 3.**
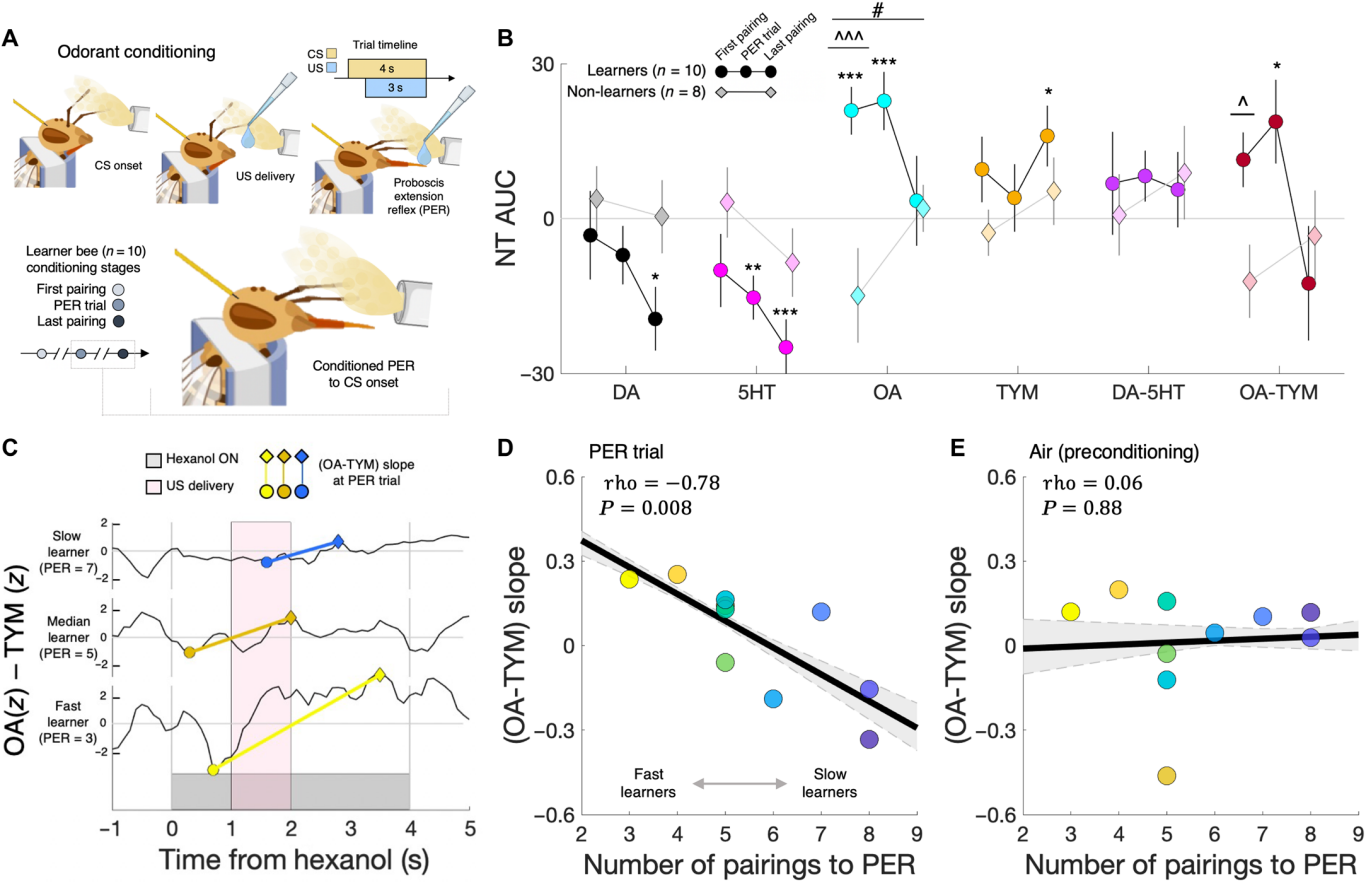
OA and TYM dynamics during odorant conditioning reflect PER learning rate. (**A**) Schematic of honey bee odorant conditioning paradigm. In each trial, bees were exposed to 4 s of hexanol, with 1.5 M sucrose delivered ~1 s after hexanol. Each trial was separated by 2-min ITI. (**B**) Response levels (AUCs) for neurotransmitters and opponent pairs for the first and last CS:US pairings and, for learners, the CS:US pairing at which a PER was first shown (PER trial). Circles and diamonds represent mean group values; vertical lines depict SEM. *P < 0.05; **P < 0.01; ***P < 0.005, one-sample *t* test. ^*P* < 0.05; ^^^*P* < 0.005, two-sample *t* test. #*P* < 0.05, paired *t* test. (**C**) Example of slow, median, and fast learner bees (*n* = 10) in vivo (OA-TYM) opponent signal slope at their PER trial. (**D**) Correlation of individual learner bees’ (OA-TYM) PER trial slope and their learning rate (trials to PER). (**E**) Correlation of learner bees’ (OA-TYM) slope during control air puff (preconditioning) with their learning rate. In both (D) and (E), the black line indicates the group-level correlation (rho), and the gray dashed lines and shaded region indicate the maximum and minimum of the (*k*-fold) bootstrapped group-level correlation.

In light of our findings that neurotransmitter response patterns to odors preconditioning were predictive of subsequent PER learning rate, we sought to quantify the adaptations in neurotransmitter responses in learners and non-learners across conditioning. We investigated differences in monoamine dynamics in learners and non-learners by specifically focusing on neurotransmitter changes in two temporal contexts: first, across conditioning stages, defined as the first odor:sucrose pairing, the trial of the first conditioned PER response (PER trial; after three to eight pairings), and the last odor:sucrose pairing (typically one to three pairings after PER trial; Fig. 3 and figs. S8 to S10); and second, across the first six odor:sucrose pairings, as all bees had at least six pairings (fig. S11). Across conditioning stages, linear modeling revealed significant main effects of group on response levels for DA, 5HT, OA, and TYM (Fig. 3B and table S3). For the learners, progressing from the first pairing to the PER trial and to the last pairing, we observed DA and 5HT decreased, and OA and TYM demonstrated nonmonotonic adaptations. Similar to our findings from the preconditioning experiments, linear modeling indicated that the differences from the first to the last pairing between learners and non-learners were specific to OA and the OA-TYM responses (table S3) [ANOVA main effect of group:pairing interaction: OA, *F*(1,33) = 6.3, *P* = 0.017; OA-TYM, *F*(1,33) = 4.0, *P* = 0.053], with the largest differences at the start of learning (first odor-sucrose pairing). The representational similarity of neurotransmitter responses across the first six odor:sucrose pairings (fig. S11) revealed that a given learners’ neurotransmitter response pattern at the first pairing was maintained across the initial pairings until the PER had been acquired, around pairings 4 to 5 on average, after which the neurotransmitter response patterns were no longer significantly correlated with their initial pattern. In comparison, no such changes in neurotransmitter response vectors were observed for non-learners. Given that DA and 5HT response levels in learners decreased steadily across conditioning stages (Fig. 3B), the shift in neurotransmitter representational similarity following PER learning appears related to OA and TYM signaling, with these neurotransmitters demonstrating significant changes in response levels before versus after the PER conditioned response (Fig. 3B).

To investigate the relation of learner bee monoamine response patterns across conditioning stages and individual bee PER learning rate, we quantified the maximum and minimum neurotransmitter responses within the first and second half of each odor:sucrose pairing trial (i.e., roughly before and after sucrose delivery) to give a measure of the rate of change (slope) of each neurotransmitter and opponent pair response across conditioning stages (fig. S12; see Materials and Methods). Given the importance of temporal features of learners’ OA-TYM responses to hexanol preconditioning in predicting the PER learning rate (e.g., step-change onset and size; fig. S5), and the gradually changing similarity in learners’ neurotransmitter response patterns from trial to trial (fig. S11), this slope metric is meant to capture individual differences in the magnitude of adaptations in neurotransmitter time series that occur across repeated odor:sucrose pairings. We found that the rate of change of the OA-TYM opponency signal during the first conditioned PER trial was significantly correlated with PER learning rate (Fig. 3, C and D, and fig. S12A), which was specific to odorant conditioning and not observed for preconditioning air puff (Fig. 3E). In addition, the rate of change of the OA-TYM opponent signal at the PER trial was highly correlated with the timing of the OA-TYM response to odor exposure preconditioning (fig. S12B), further highlighting a possible role for the temporal dynamics of OA and TYM signaling in establishing olfactory conditioning sensitivity.

## DISCUSSION

Leveraging a machine learning–enhanced approach to concurrent monoamine estimation, we were able to record for the first time the dynamics of DA, 5HT, OA, and TYM during odorant conditioning in honey bees. We focused on honey bees that showed a strong conditioned response to a series of odorant-sucrose pairings and observed, as expected from prior work (*30*), variability in the number of pairings required for a PER to develop (i.e., PER learning rate). In bees that demonstrated a PER (learner bees), we found that opponent signaling between OA and TYM on the first presentation of the odorant predicted individual bees’ PER learning rate. Overall, the results of the present paper are consistent with the roles that OA and TYM play in insect behavioral plasticity and add context to their roles as the invertebrate analogs to the mammalian epinephrine and norepinephrine (*31*). TYM is the direct precursor to OA, with TYM converted to OA in a single enzymatic step. Thus, the same modulatory neurons, e.g., the VUM neuron (*7*), make, and likely release, both transmitters in ratios (*9*, *10*), such as in the fruit fly, where the ratio of OA to TYM changes with nutrient state to regulate feeding-related locomotion (*32*). Both modulators also have opponent effects on the attractive and repulsive behaviors related to phase change in locusts (*33*) and on the flight pattern generator in moths (*34*). Our findings suggest strongly that opponency profiles at least partially determine “signaling types” that relate to whether and how quickly a honey bee forager responds to new information. Future research could relate this trait to individual bees acting to build more stable statistical models of nectar predictors (slower learners) versus bees more tuned to exploiting current models (faster learners). Such differences across foragers within a colony, which is determined in part by heritable differences among workers (*4*, *23*), could allow the colony to more adaptively respond to rapid changes in resource distributions (*35*).

The ability to record vectors of neurotransmitter dynamics will now enable a more thorough evaluation of how biogenic amines are involved in different forms of learning and more generally in regulation of behavior. Here, we have used individual differences in learning rates across bees, commonly observed in PER conditioning (*4*, *36*), to test hypotheses about how individual and opponent monoamine signaling can be associated with this behavioral distinction. Further experiments are now possible and warranted using different types of treatments to test how signaling is associated with determining learning-related states or signals of reinforcement directly. These treatments include, for example, presentation of odor (*37*) or sucrose (*38*) alone, contrasting backward and forward pairing (*39*), use of an explicitly unpaired procedure (*40*), and treatments that produce prediction errors (*41*), all of which produce different types of nonassociative, excitatory, or inhibitory learning.

Our work also parallels recent findings in mammals that opponent signaling is important for regulating plasticity in the brain. A recent experiment in rodents strongly suggests that DA and 5HT opponency acts as a critical and composite learning signal (*27*). Although the opponency system we describe here prepares a bee for learning new information, we cannot at this point confirm which monoamines, or combinations of them, represent reinforcement, which has been reported to be OA in honey bees (*7*) and DA in fruit flies (*42*). We observed that DA and 5HT response levels in both learners and non-learners decreased linearly across conditioning stages, resulting in an opponency signal not different from zero. These decreases in DA and 5-HT hint at a role for these monoamines in associative conditioning that will need to be a focus of future research; however, this role appears not to be in opponency signaling like that observed for OA and TYM, which distinguished learners from non-learners in our learning paradigm. Prior work implicating DA in aversive conditioning and punishment signaling in honey bees (*43*) suggests that changes we show here could be seen as a general state-value signal that becomes increasingly positive (i.e., reduction in aversion) across repeated odorant:sucrose pairings during PER conditioning. For OA and TYM, the response levels across learners appeared to change most significantly before versus after the conditioned PER behavior, showing concave and convex patterns across conditioning stages, respectively. As a result, the OA-TYM response related to PER learning rate started positive before the onset of conditioning, remained positive up to the PER trial, and then flipped to negative. Given the functionally antagonistic roles of OA and TYM in behavioral plasticity (*17*, *31*–*34*), this trajectory of the OA-TYM response could reflect decreased (OA-driven) appetitive conditioning and increased (TYM-driven) inhibitory conditioning after the PER was learned, thereby limiting subsequent associative learning (e.g., latent inhibition). In this way, each bee’s OA-TYM signaling type could be tested as not just phenotypically setting a bee’s learning rate preconditioning but also regulating explore-exploit choice policies during foraging via differential expression of sensitization and habituation processes. Ultimately, while further experiments are required to address these outstanding questions, our results are important steps toward a more comprehensive analysis of the roles of OA, TYM, DA, and 5HT in driving attention and reinforcement learning across species.

## MATERIALS AND METHODS

### Animals

Honey bees (*Apis mellifera*) were raised in the Arizona State University (ASU) apiary. The initial experiments were conducted at Virginia Tech (VT), with two subsequent experiments conducted at ASU. For the initial VT experiments, a large number of newly emerged (<1 day old) worker bees (female) were collected from frames in an incubator, distinctively marked, and cofostered in a common colony; at 20 days old, bees were collected from the colony entrance and transported to the VT laboratory in a cage with food ad libitum. For the ASU experiments, worker bees were marked for age in the same way and collected at the entrance of a nucleus hive. Each bee was chilled at 4°C and placed in a plastic harness. Before the surgery for neurochemical recordings, we tested bees for motivation by stimulating their antenna with 1.5 M sucrose; if bees extended their proboscis, they were used in the experiments. Of the 10 bees that learned in the PER procedure, five were collected at VT and the other five at ASU; of the eight bees that failed to learn, one was collected at VT and the other seven at ASU. Bees were recorded in the fed state, and neurochemical recording experiments were conducted throughout morning and afternoon hours.

### Neurochemical recording electrodes

Monoamine neurotransmitter recordings were taken from the antennal lobe using in-house–made carbon fiber electrodes that were adapted from those described by Kishida *et al.* (*44*). Briefly, these electrodes were made by threading a carbon fiber into a small glass capillary [90 μm outer diameter (OD); Molex]. The carbon fiber was then pulled and epoxied such that only ~150 μm (approximate length of dorsal-ventral axis in a bee) was exposed on one end of the capillary and the carbon fiber at the other end of the capillary was trimmed to ~2 mm. The ~2-mm end of the small capillary was then placed into a larger capillary (440 μm OD; Molex no. 1068450450) containing a Pt/Ir wire such that the carbon fiber overlapped the Pt/Ir wire by ~1 mm. Silver paint was used to marry the carbon fiber and Pt/Ir wire to enhance electrical conductance. The silver paint was drawn into the large capillary via capillary action before placing the small capillary inside. After a 24-hour curing period, the large and small capillaries were then epoxied together using marine epoxy. A gold pin was then soldered on to the Pt/Ir wire exposed on the other end of the large capillary. Some of the gold pin and some of the large capillary were then covered with shrink wrap, and epoxy was used to seal the joint. A stainless-steel wire with a gold pin soldered on one end was used as the reference electrode. Each carbon fiber electrode used in the in vitro and in vivo recordings in the manuscript was chosen based on their performance metrics (i.e., impedance, current response shape, RMSE values for out-of-sample predictions) that we use as standard quantitative assays for a probe’s proper functioning.

### Neurochemical recording equipment and procedures

We obtained voltammetry data in bees using the carbon fiber microelectrodes described above. The voltammetry protocol was based on previous work in humans (*11*–*15*, *44*) and rodents (*45*, *46*). The voltage forcing function used was a standard triangular voltage shape repeated at 10 Hz [ramp up from −0.6 to +1.4 V at 400 V/s, and ramp down from +1.4 to −0.6 V at 400 V/s (10 ms), then hold at −0.6 V for 90 ms]. The current response was recorded at a sampling rate of 100 kHz. We used a 97-Hz precycle protocol prior to data collection to aid in microelectrode equilibration. This precycle protocol used the previously mentioned triangular voltage ramp repeated with a shorter holding period. The voltammetry recordings were made using Molecular Devices (Molecular Devices, LLC, San Jose, CA) equipment, namely, the Axon Instruments MultiClamp 700B Patch Clamp Amplifier, the Electrochemistry Headstage (CV 7B-EC; slightly modified to allow larger current measures), and the Digidata 1550B Data Acquisition System.

### Neurochemical recordings in the antennal lobe

After the bees were cooled and placed in the plastic stage, their antennae were held in place using eicosane, a low–melting point (31° to 38°C) alkane, and a hole was opened in the head capsule to expose the brain. With careful dissection, the dorsal surface of the antennal lobe was oriented upward, for convenience of inserting the electrode. The electrode was aimed at the middle point of the dorsal surface of the antennal lobe (fig. S1), with a depth not exceeding 100 μm. Thus, the recordings were obtained from T1 (dorsal tract) glomeruli, likely among glomeruli 17, 33, 42, 28, and 36 [based on (*47*)]. The in-house–made carbon fiber electrode was placed in the antennal lobe using a stereotaxic frame (Kopf Instruments, Tujunga, CA). Fast scan cyclic voltammetry (FSCV) measurements were then taken, using the equipment and protocols described above, while the bees were exposed to a conditioning procedure (described below). A transistor-transistor logic (TTL) pulse (AFG1062, Tektronix) was used to align neurochemical and behavioral events (i.e., the moment of odor presentation).

### PER conditioning protocol

Harnessed bees that responded to sucrose (see above) were put in front of an airflow and odor delivery system. A carbon fiber electrode was then placed in the antennal lobe (see above), and neurochemical measurements were taken during the following procedures. Before conditioning, bees were first presented with an air stream, 10:1 heptanol, 10:1 2-octanone, and 10:1 hexanol on separate trials (all odor presentations lasted 4 s). These odor-only trials and, in particular, the air stimulation trial, were meant to serve as controls for the conditioning trials (i.e., to aid in isolating neurochemical signatures related to learning). All of the trials, including the conditioning trials, used a 2-min intertrial interval (ITI). Bees were conditioned using 10:1 hexanol (CS) and 1.5 M sucrose (US). Hexanol was presented for 4 s, and sucrose was delivered 1 s after odor onset by gently touching the antennae to elicit proboscis extension that is followed by feeding. Once a bee extended its proboscis after the onset of hexanol (PER), it was no longer necessary to touch its antennae. After a PER was established, hexanol, octanone, and heptanol were presented on separate trials without delivering sucrose. The unreinforced trials served as catch trials to further assess learning. Bees were considered non-learners if they did not develop a PER after 12 CS-US pairings, although they always responded to the sucrose-water droplet used as reinforcement.

### Definition of a PER

The honey bee proboscis is made up of several different parts that fold together into a straw-like mouthpart that intakes fluid nectar. It is normally folded underneath the head capsule in a retracted state (Fig. 1). When sugar stimulates taste receptors on the antennae or proboscis, or when an odor that has been associated with sugar reinforcement is presented, the first response is that the mandibles open and then the proboscis is extended from underneath the head into a feeding position. Using a fixed reference, such as when the unfolding proboscis breaks the imaginary line between the tips of the open mandibles as observed head on, PER can be quantified as a binary extended-or-not variable in each trial. Here, we defined PER as a binary variable, which was coded based on experimenter visual confirmation in each trial. More degrees of freedom in the PER can be described from electromyographic recordings of head muscles that extend and move the proboscis (*48*) or by offline analysis of video (*49*). A PER can be broken down into eight different parameters that comprise four independent degrees of freedom of movement (*48*). Expression of these parameters depends on the nature of the stimulus, e.g., sucrose versus odor (e.g., conditioned odor versus a different odor of varying chemical similarity to the conditioned odor), and the feeding state of the bee (some bees respond more vigorously than others, possibly due to differences in feeding states). Although the PER can differ across bees and test stimuli, for a given bee and test stimulus, the PER measured by electromyography (EMG) or video remains fairly constant once the proboscis is extended. For that reason, the binary variable that we use is sufficient for most studies when it reveals differences across response categories, as we show here. However, future use of voltammetry could make use of more detailed measures of the PER as well as information expressed in antennal movements in response to conditioned odors.

### Machine learning electrochemical approach

#### In vivo neuromodulator estimates

We estimated in vivo neuromodulator concentrations from in vivo current traces using an ensemble of deep CNNs that were trained and tested on in vitro data. The in vitro data consisted of voltammetry current traces obtained from single 11.5-cm carbon fiber electrodes exposed to known concentrations of DA, 5HT, OA, and TYM, and pH. We modified the InceptionTime time series classification model (*50*) to perform multivariate regression. The model was coded in Python using TensorFlow (*51*) and Keras. We used equally weighted averages of in vivo concentration estimates from multiple InceptionTime models (*50*).

Specifically, the modified InceptionTime network is based on two ResNet (*52*) blocks. Each block contains three convolutional blocks, while each convolutional block is composed of four convolutional layers in parallel, each with 32 filters and with increasing kernel sizes of 1, 10, 20, and 40. The output of each of these convolutional layers is stacked together, and batch normalization and rectified linear unit (RELU) activations are applied. This output passes through a bottleneck convolutional layer with a kernel of size one, 32 filters, and whose output serves as the input for the next convolutional block. To start the implementation of the ResNet architecture, the input passes through another bottleneck convolutional layer with kernel size 1 and 32 filters and then is added to the output of the first ResNet block. After activation, this serves as the input to the next ResNet block. This operation is repeated for the second ResNet block, except that the input is now replaced by the output of the first ResNet block. Lastly, after passing through a global average pooling, the output of the second ResNet block passes through a dense layer with five output nodes; this gives the predictions of the five analytes. All activation functions are RELU, except after the last dense layer, which uses a linear activation function.

All models used the mean square error loss function. Training schedule used the ADAM optimizer (*53*), with an initial learning rate of 1 × 10^−3^ that halved after 5 epochs without decrease of validation loss. These single electrode carbon fiber models did not have a minimum learning rate. The batch size was 64, and the loss on the validation set was calculated after each epoch. The model from the epoch with the lowest validation loss was selected as the final model for that run.

Final predictions of in vivo data are generated using a mixture of experts (i.e., where we train an ensemble of models with the same hyperparameters and average their predictions). Differences in weights generated during initialization, variations in the order in which data are fed to the algorithm during training, and the stochastic descent algorithm all introduce variation to the convergence of each model. In addition, different validation sets were used in each run to avoid overfitting.

#### In vitro training data for carbon fiber bee electrodes

We collected model training data using five carbon fiber electrodes, and these electrodes were subsequently used to record in vivo current measurements in the bee antennal lobe. Five datasets were collected on each electrode, one for each analyte—DA, 5HT, OA, and TYM—and pH. The data acquisition equipment was of the same type (same component type/number) used in the in vivo data collection. Most of the four neurotransmitter (DA, 5HT, OA, and TYM) datasets were collected with 40 concentrations of the analyte dispersed at equal intervals over the range from 0 to 250 nM, with a pH of around 6.8 (approximate pH of the hemolymph), whereas the other three analytes were kept at 0 nM. The pH dataset for each electrode was collected with 11 pH values in the range 6.8 to 7.8 and with the concentration of the four neurotransmitters set to 0 nM. For each electrode, we randomized the order of neurotransmitter dataset collection, and within each neurotransmitter dataset, the order of unique concentrations was also randomized.

To collect data, each working and reference electrode was inserted vertically into a 1.5-ml Eppendorf containing the neuromodulator concentration of interest for that collection run. We then collected current data at 10 Hz for 65 s using the same measurement protocol deployed during in vivo collection. We selected the most stable continuous 15-s section from the second half of the 10-Hz 65-s time window for training to reduce variation due to electrical noise and equilibration. See (*13*) and (*16*) for (similar) previously published methods.

#### In vitro model training for carbon fiber bee electrodes

Because we do not yet have enough data collected on 11.5-cm carbon fiber electrodes for a generalized model, we create a specific model for each electrode (e.g., on electrode model). To do this, we train a mixture of experts, where the final prediction was the average of an ensemble of 20 training runs. A training run involved a dataset containing 150 sweeps from each unique concentration, which was split into a training set containing 90% of the data and a validation set containing the remaining 10%. The data were split by concentration, such that all data points of any given concentration were in the same set. Before training the model, the data were *z*-scored within the analyte and then shifted by 10 standard deviations per analyte to avoid zero gradients. After training the model, the inverse of the normalization procedure was performed on the predictions. The models were trained for 100 epochs.

#### In vitro model evaluation for carbon fiber bee electrodes

A 10-fold cross-validation test was performed for each of the 11.5-cm bee electrodes. The in vitro data were split into 10 discrete folds without overlapping values. Each fold was held out as a test set, and an ensemble of models was created via a training run on the remaining in vitro data. Ensembles of 20 models were used. The mean of the predictions from each 20-model ensemble was calculated for its corresponding test set. All test sets were combined, thus resulting in all in vitro data points being predicted from ensembles of models that had never been trained or validated on those data points (see Fig. 1B).

#### In vivo concentration estimates for carbon fiber bee electrodes

We generated in vivo concentration estimates using the same carbon fiber models described above. Because we created on-electrode models, we matched in vitro models to the carbon fiber that collected the in vivo data. In vivo current traces were differentiated as in the training and then evaluated by the specific models built for an electrode. Average predictions across this ensemble of models (mixture of experts) were used as the final concentration estimates.

### Neurochemical data analysis

#### Learning rate correlation with monoamine dynamics

Each experiment consisted of the preconditioning air and odor stimulation trials and the conditioning trials. For each bee, the model-generated monoamine neurotransmitter time series—20-s “snippets” centered on the TTL-triggered odor presentation—for every trial were first smoothed using a 500-ms lagging sliding window and then *z*-scored within trial (i.e., trial mean and standard deviation computed using the full 20-s snippet). This smoothing and normalization were conducted for each neurotransmitter individually, resulting in a four vectors of monoamine dynamics, which were further cut to a temporal window spanning from 1 s preodor to 5 s post-odor (odor is present from 0 to 4 s). We computed OA-TYM opponency and DA-5HT opponency time series by taking the difference between the respective neurotransmitter time series pairs after all four neurotransmitters had been individually preprocessed.

Neurotransmitter response levels were defined as the sum of the neurotransmitter time series within the 0- to 4-s odor presentation window (i.e., taking the AUC). We computed a measure of the rate of change of neurotransmitter time series (i.e., slope) by identifying the magnitude and timing of the maximum or minimum (extrema) of each time series in the 0- to 2-s window and 2- to 4-s window of each trial of the preconditioning and conditioning phase; the slope metric was then calculated as the difference in extrema values divided by the time between them. To determine the peak opponency response and its relation to learning rate, we identified the timing of positive clusters for each opponent time series via its zero crossings. We computed the Pearson correlation coefficient for the timing of the onset of the largest positive cluster (i.e., maximum opponent response) and the number of hexanol:sucrose pairings before reaching a PER (i.e., learning rate). We also computed the Pearson correlation coefficient between neurotransmitter response levels (AUCs) and learning rate. This analysis was conducted for all neurotransmitters individually as well as both monoamine opponent pairs, OA-TYM and DA-5HT. We computed a measure of the variation in the correlations between neurotransmitter response features and learning rate via a leave-one-out bootstrapping procedure. For this procedure, we iteratively computed the group-level correlations while holding out each bee once (i.e., *k*-fold), and we took the minimum and maximum of the resulting set of (*k* = 10) group-level correlations as depicting the variation in the group-level correlation across all learner bees.

#### Representational similarity analysis

We conducted representational similarity analyses (RSA) to characterize the covariation in patterns of neurotransmitter response levels (i.e., AUC values) for learners and non-learners during the preconditioning odor stimulation trials (fig. S4) and across conditioning trials (fig. S11).

For the preconditioning RSA, we first calculated, for each bee individually, the response level (AUC; 0- to 4-s odor window) of each neurotransmitter and opponent pair to the air and odor (hexanol, heptanol, and 2-octanone) stimulation trials (fig. S4A). This resulted in a four-by-six data matrix (odors-by-neurotransmitters) of neurotransmitter AUC values for each bee, from which we then constructed a four-by-four representational similarity matrix by computing the pairwise Pearson correlation coefficient of the rows of this data matrix. The lower triangle of this representational similarity matrix captures the covariation in neurotransmitter response patterns for the four preconditioning odor stimulation conditions. For instance, the first column of the representational similarity matrix depicts the correlation between a bee’s neurotransmitter response pattern to air stimulation and its neurotransmitter response pattern to the three odors. Looking across all bees, we tested for whether the neurotransmitter response patterns were significantly similar across air and odor conditions by performing a one-sample *t* test of bees’ (*N* = 18) Pearson correlation coefficients for each of the seven off-diagonal (lower triangle) element of the representational similarity matrix, with a null hypothesis of correlation coefficients being equal to zero, indicating no significant pairwise similarity between conditions at the group level. We performed this preconditioning RSA analysis using all bees (*N* = 18) and for learner (*n* = 10) and non-learner (*n* = 8) bees separately.

For the conditioning RSA, we computed a representational similarity matrix for each bee in a similar manner as the preconditioning RSA, though involving the first six odor:sucrose conditioning trials. Thus, for each bee, we first computed a six-by-six data matrix (trials-by-neurotransmitters) of neurotransmitter AUC values and then computed the individual and group-level representational similarity matrices that capture the covariation in neurotransmitter response patterns across the six initial odor:sucrose pairings. We chose to examine the first six trials since every bee had at least six pairings of odor:sucrose. With this setup, the first column of a bee’s representational similarity matrix depicts the correlation between the neurotransmitter response pattern to the first odor:sucrose pairing and the neurotransmitter response patterns of the ensuing pairings [elements of arrow (i) in fig. S11B]. In addition, the first lower diagonal of the representational similarity matrix (i.e., the diagonal directly below the primary diagonal) depicts the covariation in neurotransmitter response patterns from trial to trial [elements of arrow (ii) in fig. S11B]. In addition to computing the statistical significance of each off-diagonal element via one-sample *t* test, we also computed the significance of the average correlation across the elements of the first column and first lower diagonal of the representational similarity matrix using a one-sample *t* test. We conducted this conditioning RSA procedure for all bees and for learners and non-learners separately; differences between learners and non-learners were computed using a two-sample *t* test.

#### SVD analysis

We performed a data-driven reduction of honey bee monoamine dynamics using the SVD (fig. S7). The SVD analysis included all learner bees (*n* = 10) and non-learners (*n* = 8). For each bee, the DA, 5HT, OA, and TYM time series, aligned to the onset of hexanol, were concatenated length-wise, converting each bee’s four-vector of monoamine time series (4 by 61 matrix) into a one-vector (1 × 4*61). The resulting one-vector time series were concatenated vertically across all bees, forming an 18 by 244 matrix **Y**. Computing the SVD of this data matrix **Y** produces a matrix of right singular vectors **V** that represent latent monoamine patterns across bees and a matrix of left singular vectors **U** that reflect the “loading” of the latent monoamine patterns for each bee. We computed the Pearson correlation coefficients of the left singular vector loadings with the learning rate, which revealed one latent pattern, right singular vector 4, which demonstrated a significant correlation. We depicted the monoamine signals of this latent pattern, as well as the DA/5HT and OA/TYM opponent dynamics, by reconstructing the four-vector of monoamine dynamics based on this latent pattern (i.e., dividing the right singular vector 4 back into its component signals; fig. S7, C and D).

#### Statistical modeling and quantitative analysis

We performed a set of linear modeling analyses to quantify the effects of group (learner or non-learner), odor (air or hexanol), conditioning trial (first or last), and interactions among these variables on neurotransmitter response levels (i.e., AUCs). For the preconditioning phase, each linear model consisted of regressing the main effects of group, odor, and their interaction on individual neurotransmitter response levels (i.e., a single model for each neurotransmitter; table S1); we performed this analysis across all bees (*N* = 18; table S1) and for learners (*n* = 10) and non-learners (*n* = 8) separately (i.e., only including main effect of odor; table S2). Linear modeling was conducted using MATLAB’s function fitlme with subsequent ANOVA *F* tests over fixed-effects model terms using MATLAB’s function anova. We repeated this linear modeling analysis for the conditioning phase, with each model consisting of the main effects of group, conditioning trial, and their interaction (table S3). Following linear modeling analyses, we performed post hoc statistical testing of learner and non-learner neurotransmitter AUCs in response to different odors and conditioning phase, using one-sample *t* tests to assess whether AUC values were different from zero, two-sample *t* tests to assess whether AUC values were different between groups, and paired *t* tests to assess within-group differences in AUC values in response to different odors and conditioning phases.
