# Supplementary material for: Octopamine and tyramine dynamics predict learning rate phenotypes during associative conditioning in honey bees
Source: Sci Adv. 2026 Feb 11;12(7):eaea8433. doi: 10.1126/sciadv.aea8433 (PMC12893229; doi:10.1126/sciadv.aea8433)
Supplement: Supplementary file 1 — Figs. S1 to S12 Tables S1 to S3 [file sciadv.aea8433_sm.pdf]

Science Advances
AAAS

Supplementary Materials for

Octopamine and tyramine dynamics predict learning rate phenotypes during associative conditioning in honey bees

Lester P. Sands *et al.*

Corresponding author: Lester P. Sands, psands@vtc.vt.edu; Pendleton R. Montague, read@vtc.vt.edu; Brian H. Smith, brianhsmith@asu.edu

Sci. Adv. **12**, eaea8433 (2026)
DOI: 10.1126/sciadv.aea8433

This PDF file includes:

Figs. S1 to S12
Tables S1 to S3

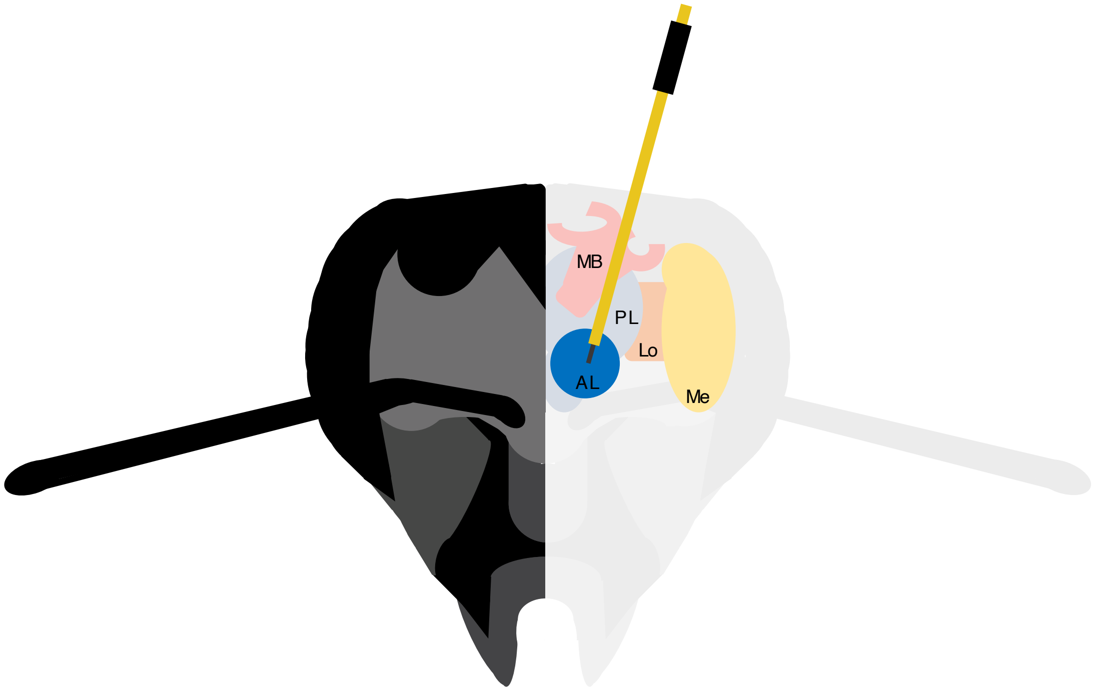

SI Appendix Figure S1. Carbon fiber electrode placement in honey bee antennal lobe. Cartoon depiction of electdoe insertion and neurochemical recording site within the antennal lobe (AL), shown relative to other portions of honeybee neuroanatomy; MB = mushroom body, PL = protocerebral lobe, Lo = lobula, Me = medulla.

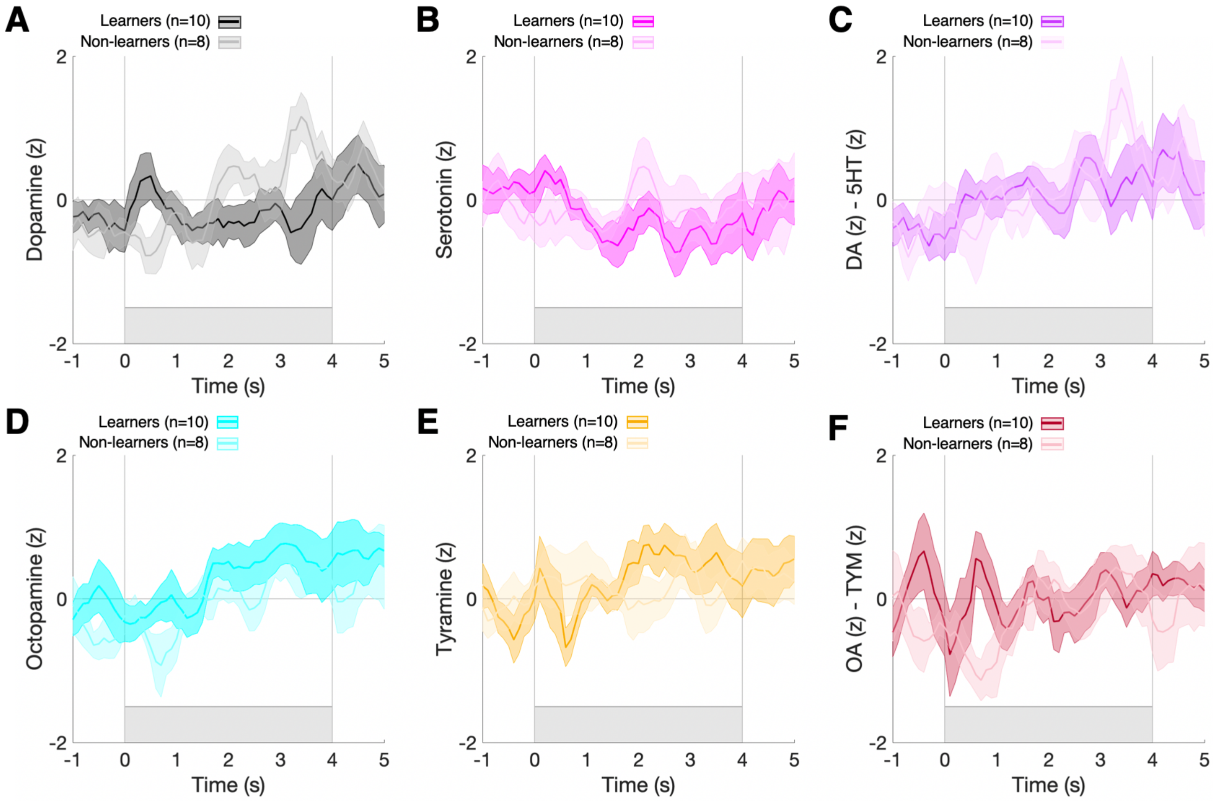

SI Appendix Figure S2. Learner and non-learner bee neurotransmitter response to air puff exposure pre-conditioning. Group-average in-vivo neurotransmitter and opponent pair time series (10 Hz) in response to air puff pre-conditioning for learner bees (n=10) and non-learner bees (n=8). Shaded regions indicate standard error of the mean (SEM), and the grey vertical lines and shaded rectangle indicate the air puff temporal window.

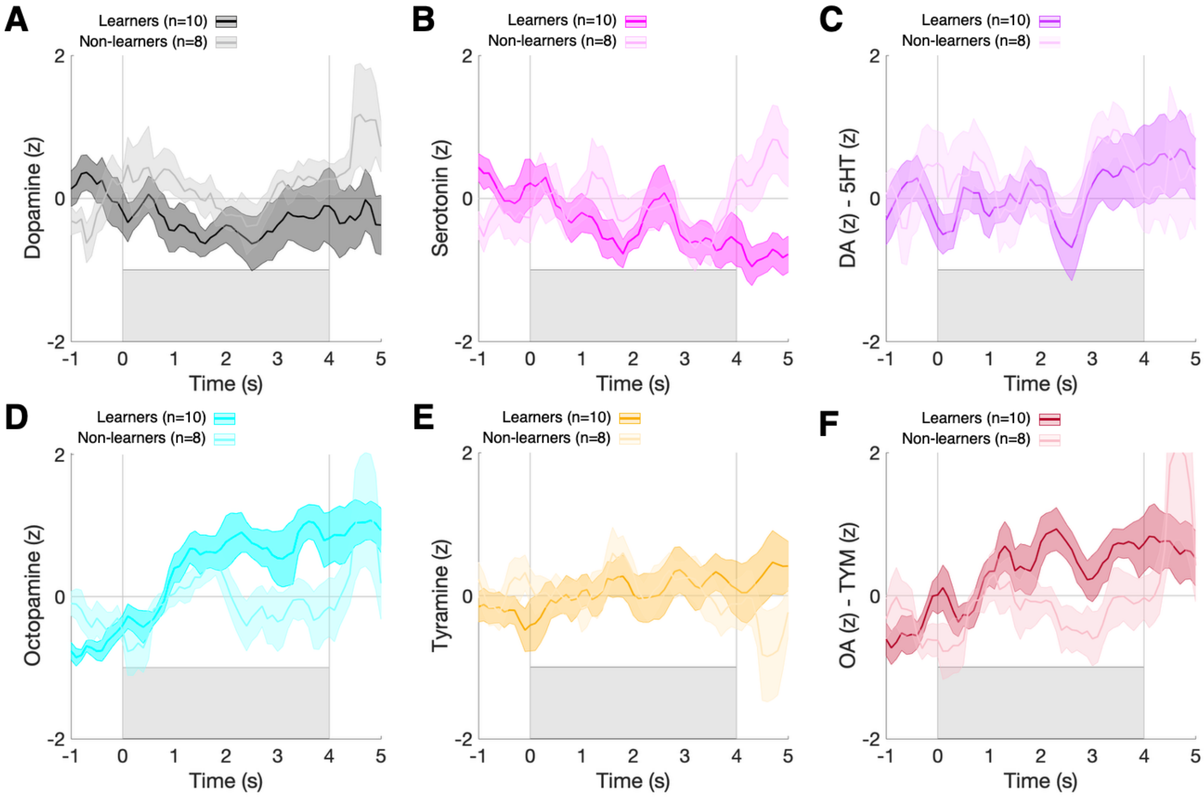

SI Appendix Figure S3. Learner and non-learner bee neurotransmitter response to hexanol exposure pre-conditioning. Average in-vivo neurotransmitter and opponent pair time series in response to hexanol pre-conditioning for learner bees (n=10) and non-learner bees (n=8). Shaded regions indicate standard error of the mean (SEM), and the grey vertical lines and shaded rectangle indicate the hexanol temporal window.

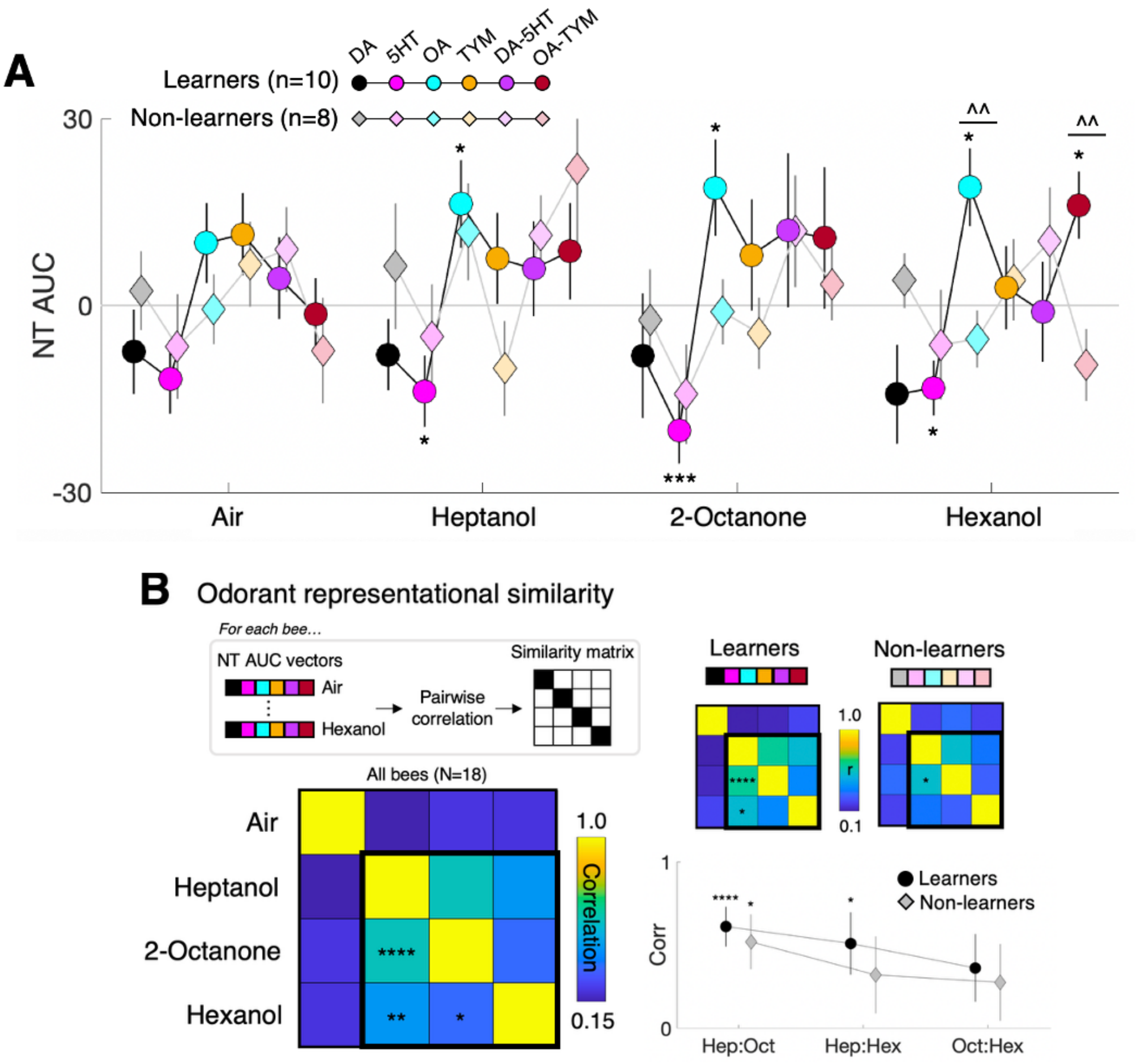

SI Appendix Figure S4. Learner and non-learner pre-conditioning odorant responses across neurotransmitters within antennal lobe. (A) Response levels (AUCs) for neurotransmitters and opponent pairs across the air and odor stimulation conditions. Circles and diamonds represent mean group-level AUC values, and the vertical lines depict SEM. *p<0.05,***p<0.005 one-sample t-test; ^^p<0.01 two-sample t-test. **(B)** Representational similarity analysis of neurotransmitter response patterns (vector of NT AUC values) to air, heptanol, 2-octanone, and hexanol exposure. Response levels (AUC) for DA, 5HT, OA, TYM, (DA-5HT), and (OA-TYM) time series in the 4-second odor presentation were correlated across all bees (left; N=18) as well as for learners and non-learners separately (right). Note that the matrices and color bars depict the random-effects representational similarity (i.e., computed for each bee individually). *p<0.05, **p<0.01, ****p<0.001 one-sample t-test.

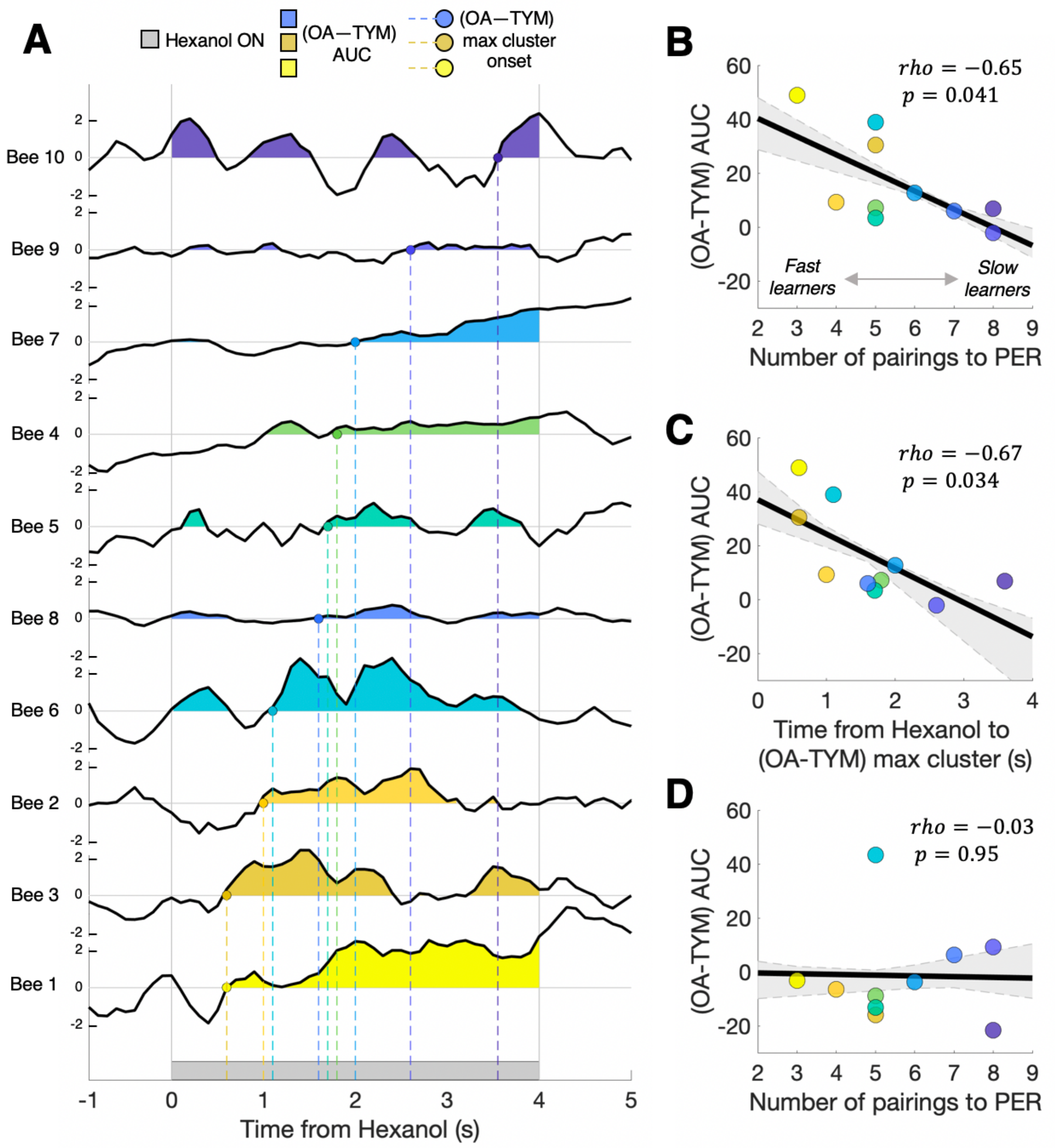

SI Appendix Figure S5. Learner bee (OA-TYM) response to first hexanol exposure pre-conditioning. (A) Individual (n=10) learner bees in-vivo (OA-TYM) opponent response at the first hexanol exposure for each bee. Shaded regions indicate positive area under the curve, and the onset of the maximum positive cluster is indicated by the colored circles and dashed lines. **(B)** Correlation of individual learner bees' (OA-TYM) AUC during hexanol exposure and their subsequent learning rate (trials to PER) during conditioning. **(C)** Correlation of individual learner bees' (OA-TYM) AUC and (OA-TYM) max positive cluster onset time during hexanol exposure. **(D)** Correlation of learner bees' (OA-TYM) AUC values during control air puff with their learning rate. In **(B-D)**, dashed lines and shaded region indicate the maximum and minimum of the k-fold bootstrapped correlation across learner bees.

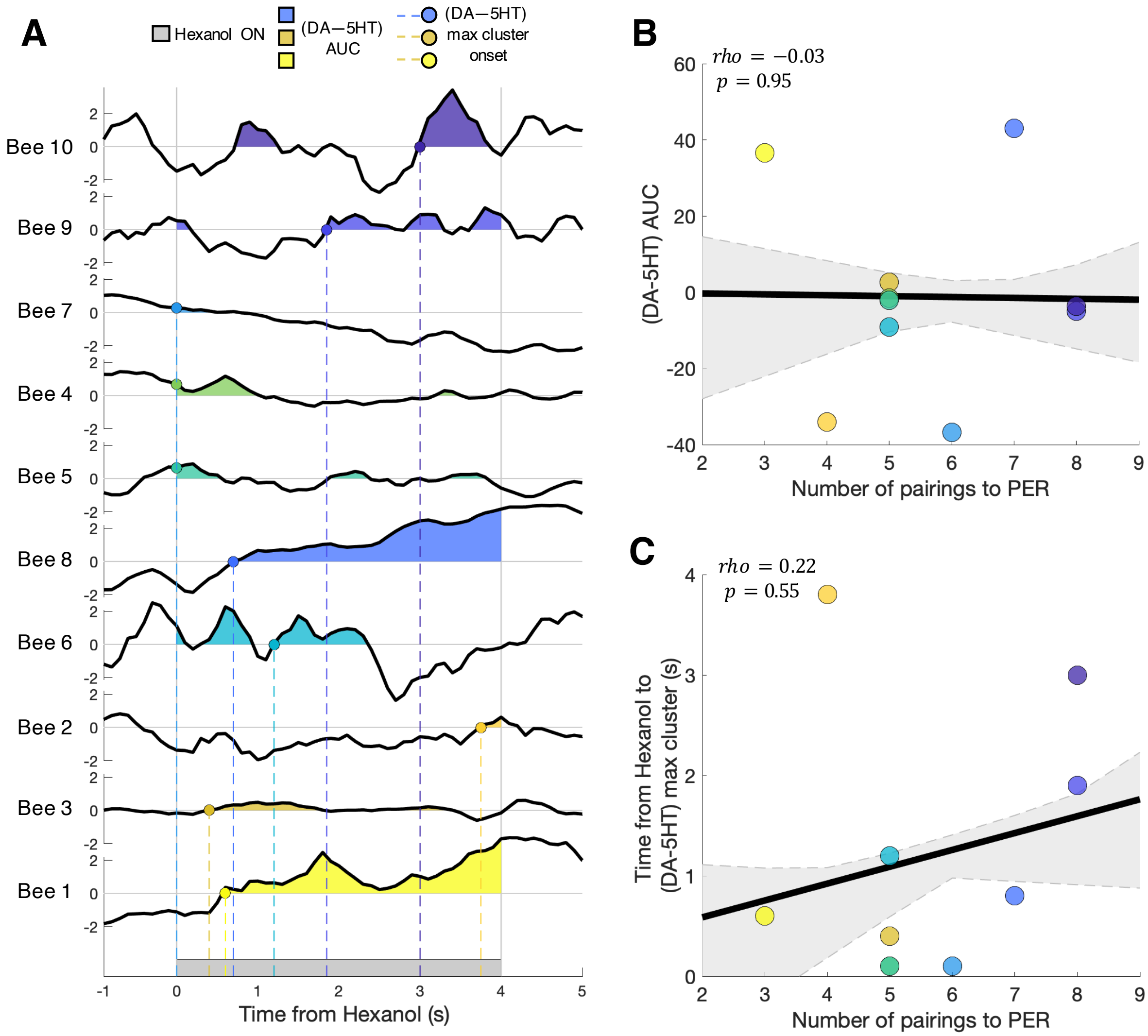

SI Appendix Figure S6. Learner bee (DA–5HT) response to first hexanol exposure pre-conditioning. (A) Individual (n=10) learner bees in-vivo (DA-5HT) opponent response at the first hexanol exposure for each bee. Shaded regions indicate positive area under the curve, and the onset of the maximum positive cluster is indicated by the colored circles and dashed lines. **(B)** Correlation of individual learner bees' (DA–5HT) AUC during hexanol exposure and their subsequent learning rate (trials to PER) during conditioning. **(C)** Correlation of individual learner bees' (DA–TYM) max positive cluster onset time during hexanol exposure with their subsequent learning rate (trials to PER) during conditioning. In **(B,C)**, black line depicts the group-level correlation (rho), and the grey dashed lines and shaded region indicate the maximum and minimum of the k-fold bootstrapped correlation across learner bees.

22

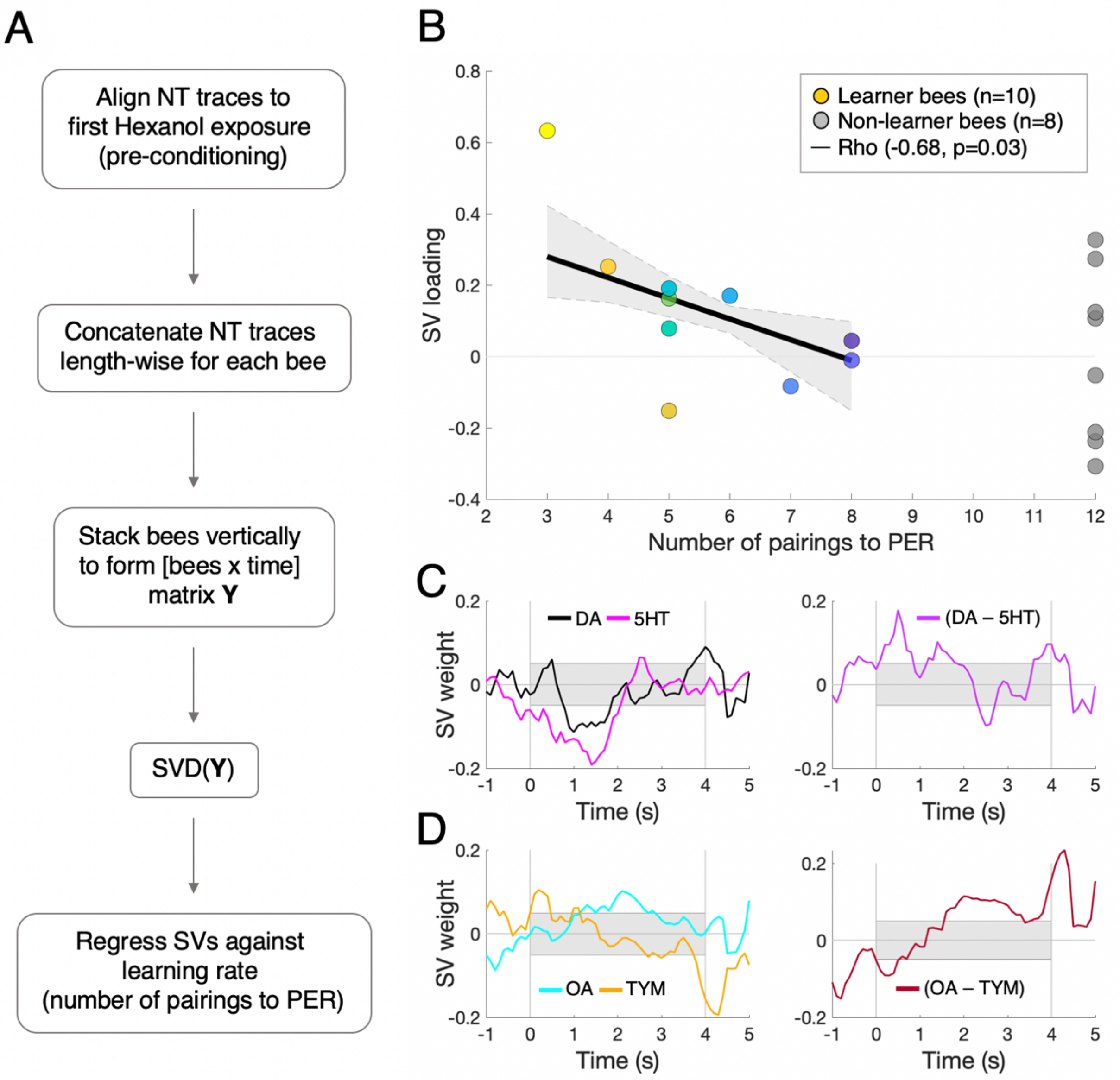

SI Appendix Figure S7. Singular value decomposition analysis. (A) Summary of SVD analysis steps (also see Methods). **(B)** Correlation of singular vector 4 loadings with learning rate (number of pairings to PER). **(C)** Latent pattern of dopamine (black) and serotonin (magenta) time series (left) and opponent response (right). Grey bar depicts presence of hexanol. **(D)** Same as **(C)** but for octopamine (cyan) and tyramine (orange).

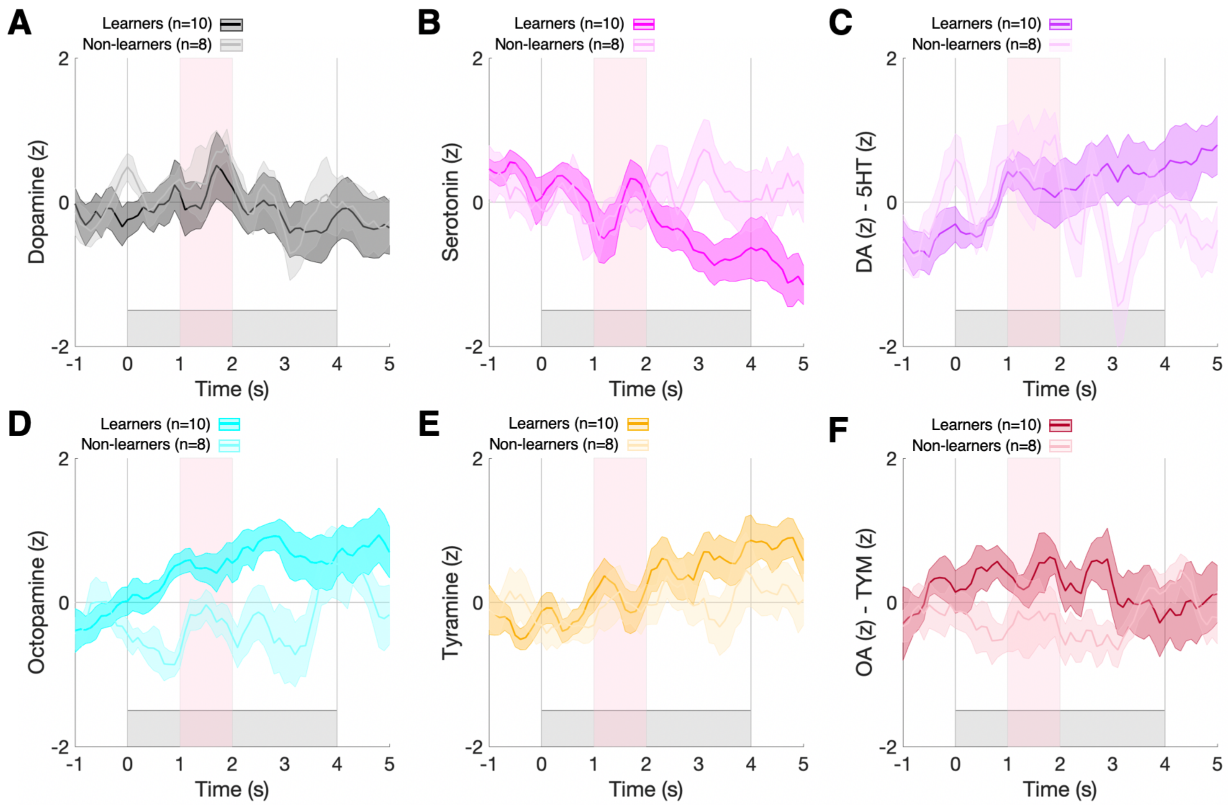

SI Appendix Figure S8. Learner and non-learner bee neurotransmitter response to first hexanol:sucrose pairing. Average in-vivo neurotransmitter and opponent pair time series in response to the first hexanol:sucrose pairing during conditioning for learner bees (n=10) and non-learner bees (n=8). Shaded regions indicate standard error of the mean (SEM), and dashed lines indicate the air puff temporal window.

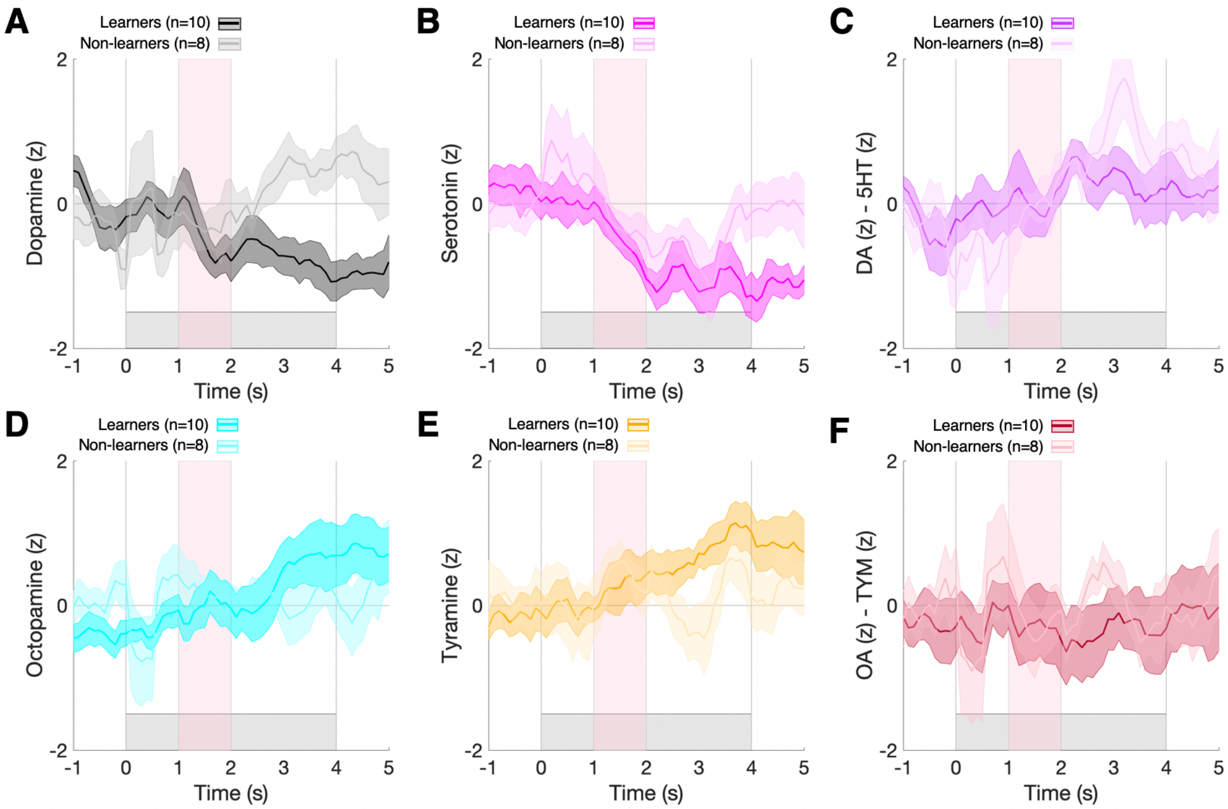

SI Appendix Figure S9. Learner and non-learner bee neurotransmitter response to last hexanol:sucrose pairing. Average in-vivo neurotransmitter and opponent pair time series in response to last hexanol:sucrose pairing during conditioning for learner bees (n=10) and non-learner bees (n=8). Shaded regions indicate standard error of the mean (SEM), and dashed lines indicate the air puff temporal window.

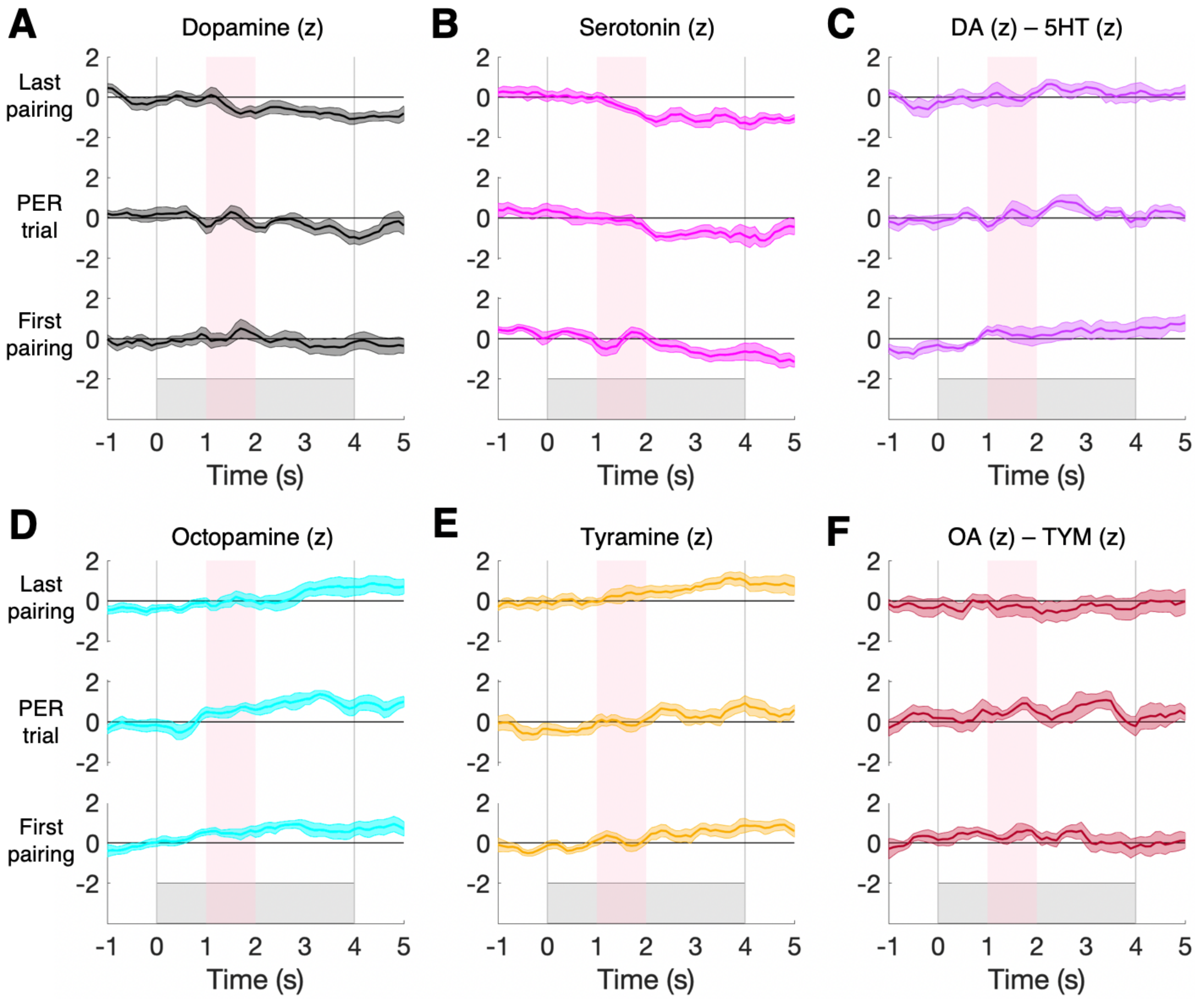

SI Appendix Figure S10. Learner bee neurotransmitter responses across conditioning. Average in-vivo neurotransmitter and opponent pair time series in response to first hexanol:sucrose pairing, the PER trial, and the last hexanol:sucrose pairing during conditioning for learner bees (n=10). Shaded regions of time series indicate standard error of the mean (SEM); grey shaded region and vertical lines indicate the hexanol temporal window; the pink shaded region indicates sucrose delivery temporal window.

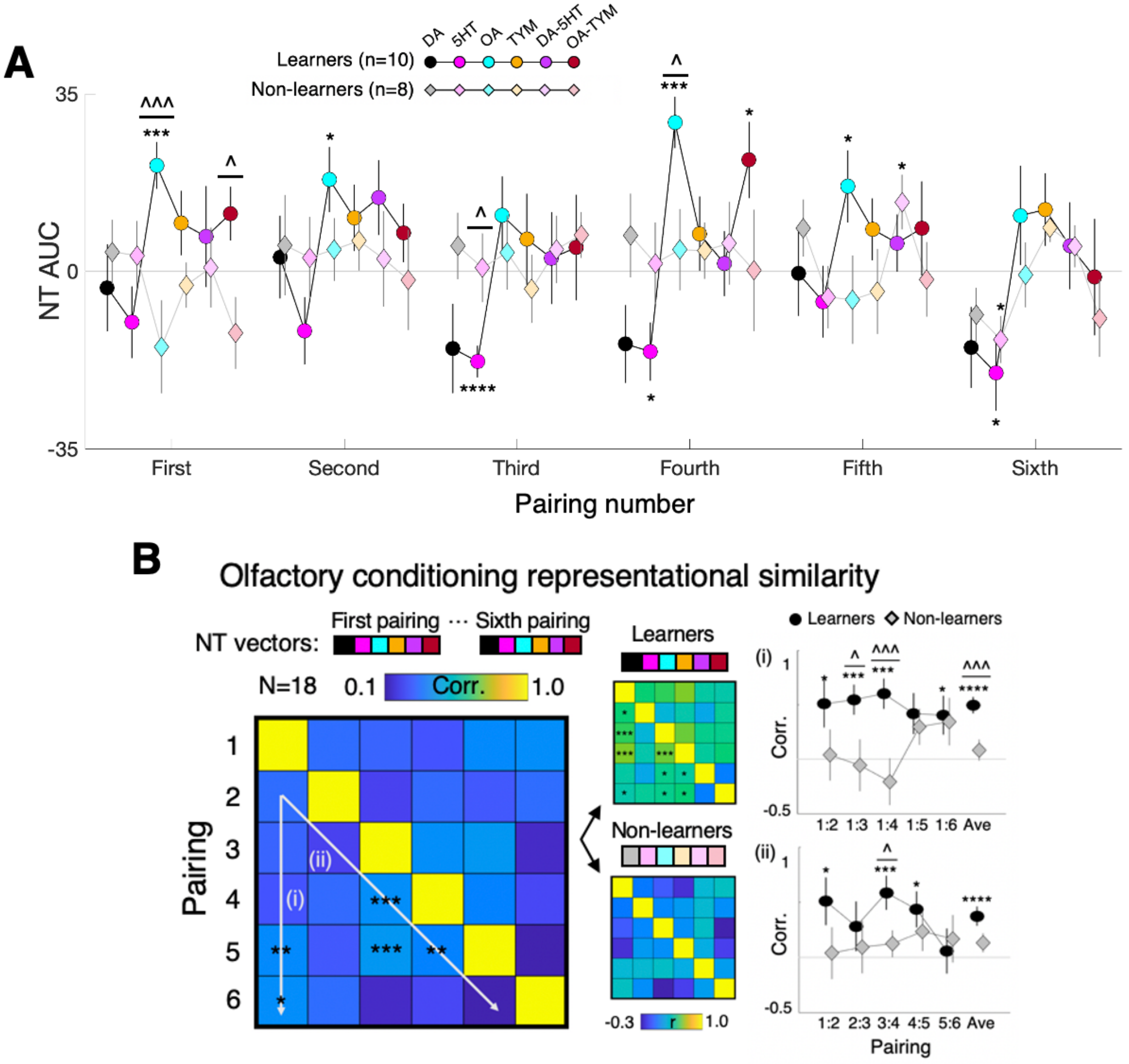

SI Appendix Figure S11. Learner and non-learner odorant conditioning responses across neurotransmitters within antennal lobe. (A) Response levels (AUCs) for neurotransmitters and opponent pairs for the first six CS:US pairings for learners and non-learners. Circles and diamonds represent mean group values; vertical lines depict SEM. *p<0.05, **p<0.01, ***p<0.005. ****p<0.001 one-sample t-test; ^p<0.05, ^^^p<0.005 two-sample t-test. **(B)** Representational similarity analysis of neurotransmitter response patterns to the first six CS:US pairings. Response levels (AUC) for DA, 5HT, OA, TYM, (DA-5HT), and (OA-TYM) time series in the 4-second odor presentation were correlated across all bees (left; N=18) and (right) for learners and non-learners separately. Note that the matrices and color bars depict the mixed-effects representational similarity (i.e., computed mean NT vectors for each bee individually). *p<0.05, **p<0.01, ***p<0.005, ****p<0.001 one-sample t-test. ^p<0.05, ^^^p<0.005 two-sample t-test.

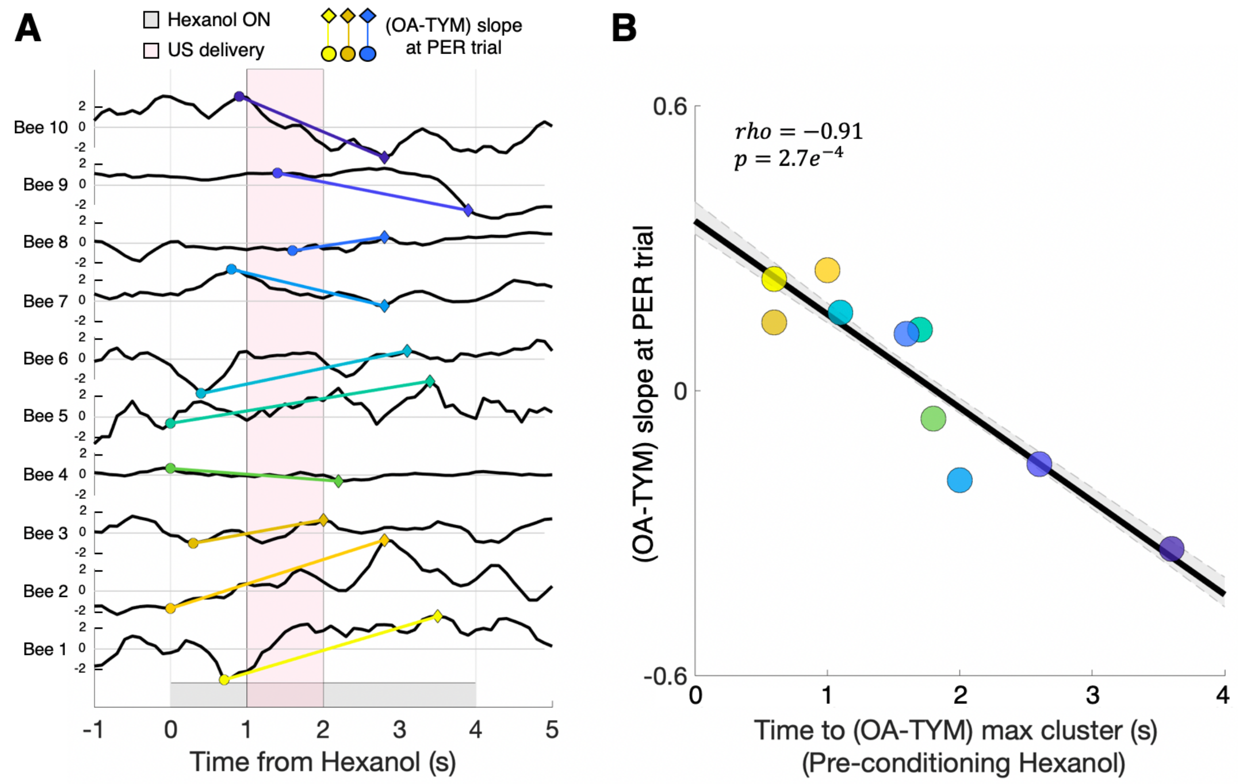

SI Appendix Figure S12. Learner bee (OA-TYM) rate of change at PER trial during odorant conditioning. (A) Individual (n=10) learner bees in-vivo (OA-TYM) opponent response at the PER trial for each bee. Slopes are computed based on the extreme (max or min) response values in the first (0-2s; circles) and second half (2-4s; diamonds) of hexanol exposure. **(B)** Correlation of individual learner bees' (OA-TYM) slope at PER trial and their pre-conditioning hexanol exposure (OA-TYM) max cluster onset timing. The dashed lines and shaded region indicate the maximum and minimum of the k-fold bootstrapped correlation across learner bees.

NT AUC ~ Group + Odor + Group:Odor						
**Model**	**Group**		**Odor**		**Interaction**	
	F(1,33)	p-value	F(1,33)	p-value	F(1,33)	p-value
DA	**5.27**	**0.028**	0.16	0.69	0.42	0.52
5HT	1.36	0.25	0.01	0.94	0.01	0.90
OA	**10.1**	**0.003**	0.12	0.73	1.3	0.26
TYM	0.22	0.63	0.69	0.41	0.21	0.65
DA–5HT	0.82	0.37	0.07	0.78	0.20	0.65
OA–TYM	**6.8**	**0.013**	1.6	0.21	2.7	0.11

SI Appendix Table S1. Linear model of pre-conditioning neurotransmitter responses to air and hexanol in all bees. The equation of the linear model is shown in the first row, with the ANOVA marginal statistical test results shown for each main effect of the model (group, odor) and their interaction between group and odor. For each ANOVA, the degrees of freedom are shown for the F-statistic.

	**Learner NT AUC ~ Odor**		**Non-learner NT AUC ~ Odor**	
**Model**	**Odor**		**Odor**	
	F(1,19)	p-value	F(1,15)	p-value
DA	0.38	0.54	0.05	0.83
5HT	0.03	0.87	6.3e-4	0.98
OA	0.69	0.41	0.48	0.49
TYM	0.82	0.37	0.07	0.78
DA–5HT	0.29	0.59	0.01	0.91
OA–TYM	**4.57**	**0.045**	0.05	0.83

SI Appendix Table S2. Linear model of pre-conditioning neurotransmitter responses to air and hexanol in learners and non-learners. The equation of the linear model is shown in the first row, with the ANOVA marginal statistical test results shown for each main effect of the model (group, odor) and their interaction. For each ANOVA, the degrees of freedom are shown for the F-statistic.

NT AUC ~ Group + Pairing + Group:Pairing						
**Model**	**Group**		**Pairing**		**Interaction**	
	F(1,33)	p-value	F(1,33)	p-value	F(1,33)	p-value
DA	**4.26**	**0.047**	1.94	0.17	0.81	0.37
5HT	**5.94**	**0.020**	3.55	0.07	0.05	0.82
OA	**8.06**	**0.008**	0.002	0.97	**6.32**	**0.017**
TYM	**4.58**	**0.039**	1.4	0.25	0.02	0.89
DA–5HT	0.098	0.75	0.17	0.68	0.31	0.58
OA–TYM	0.59	0.45	0.85	0.36	4.03	0.053

SI Appendix Table S3. Linear model of neurotransmitter responses to first and last pairing during conditioning in all bees. The equation of the linear model is shown in the first row, with the ANOVA marginal statistical test results shown for each main effect of the model (group, pairing) and their interaction. For each ANOVA, the degrees of freedom are shown for the F-statistic.
